# Climate variability, animal reservoir and transmission of scrub typhus in Southern China

**DOI:** 10.1371/journal.pntd.0005447

**Published:** 2017-03-08

**Authors:** Yuehong Wei, Yong Huang, Xiaoning Li, Yu Ma, Xia Tao, Xinwei Wu, Zhicong Yang

**Affiliations:** Guangzhou Center for Disease Control and Prevention, Guangzhou, Guangdong Province, China; University of Tennessee, UNITED STATES

## Abstract

**Objectives:**

We aimed to evaluate the relationships between climate variability, animal reservoirs and scrub typhus incidence in Southern China.

**Methods:**

We obtained data on scrub typhus cases in Guangzhou every month from 2006 to 2014 from the Chinese communicable disease network. Time-series Poisson regression models and distributed lag nonlinear models (DLNM) were used to evaluate the relationship between risk factors and scrub typhus.

**Results:**

Wavelet analysis found the incidence of scrub typhus cycled with a period of approximately 8–12 months and long-term trends with a period of approximately 24–36 months. The DLNM model shows that relative humidity, rainfall, DTR, MEI and rodent density were associated with the incidence of scrub typhus.

**Conclusions:**

Our findings suggest that the incidence scrub typhus has two main temporal cycles. Determining the reason for this trend and how it can be used for disease control and prevention requires additional research. The transmission of scrub typhus is highly dependent on climate factors and rodent density, both of which should be considered in prevention and control strategies for scrub typhus.

## Author summary

Scrub typhus has been endemic in southern China for several decades. In recent years, it has been increasingly reported and has become a significant health concern in China. The incidence of scrub typhus, a vector-borne disease, is influenced by the density of rats and changes in climate. Several studies have focused on the influence of climate and rat density on scrub typhus independent of one another; however, few studies investigate such factors simultaneously. Furthermore, global climate events such as El Niño have not been considered in any study of scrub typhus risk factors. This study reports novel factor research of scrub typhus in southern China. Data of climate, rat density and cases were collected on a monthly basis. Time-series Poisson regression models and distributed lag nonlinear models (DLNM) were used to evaluate the relationship between risk factors and scrub typhus. Finally, relative humidity, rainfall, DTR, MEI and rodent density were identified as risk factors of the incidence of scrub typhus.

## Introduction

Scrub typhus, also known as tsutsugamushi disease, is a rickettsial disease that is highly endemic in East Asia and the western Pacific Ocean areas [1]. In recent years, scrub typhus has been increasingly reported and has become a significant health concern in China[2–5]. Currently, scrub typhus cases are prevalent in areas of high population density in southern China, indicating that millions of people are at risk of contracting scrub typhus[3].

Scrub typhus is caused by infection with *Orientia tsutsugamushi*, which is often carried by the rodent hosts *Rattus flavipectus*, *Rattus rattoides*, *Apodemus agrarius*, and *Suncus murinus* [6, 7]. Transmission of *Orientia tsutsugamushi* to humans occurs through the bite of infected trombiculid mite larvae of the genus *Leptotrombidium* (chiggers). The incubation period is approximately 4–21 days, and the typical clinical symptoms include fever, eschars or ulcers, lymphadenopathy, and skin rash [8]. It predominantly occurs in farmers but also occurs in urban populations through exposures in parks and visits to the country side [9].

Transmission of scrub typhus varies across seasons and geographical areas in China. The rate of transmission is thought to be influenced by rodent population, percentage of rodents infected with *O*. *tsutsugamushi*, contact frequency between rodents and humans and chigger abundance [10, 11]. The incidence is also thought to be influenced by climate[12]. For example, high temperature and rainfall cause an increase in chigger abundance, increasing the transmission of scrub typhus[11]. The El Niño Southern Oscillation (ENSO) has been linked to increased rodent populations, which were associated with rodent-borne diseases such as plague, and hantavirus pulmonary syndrome (HPS) [13–15]. The same effects might apply to the scrub typhus transmission but have not yet been reported.

These environmental variables have been widely used as indicators for the risk of transmission of other rodent-borne diseases[16–18]. However, research exploring the connection between scrub typhus and climate variables is sparse. Limited studies have demonstrated associations between rodent population density, climate variability (such as temperature, rainfall, relative humidity), ENSO and the transmission rate of scrub typhus. Furthermore, the quantitative relationship between climate variation and the transmission rate of scrub typhus remains to be determined, especially in southern China, where scrub typhus incidence is the highest in the country.

We aimed to examine the potential impact of rodent density and various climatic variables on the transmission rate of scrub typhus while accounting for lag time. We also aimed to provide strategic insights into the current and future impact of climate change on scrub typhus transmission, especially in subtropical regions.

## Methods

### Study location

**Fig 1:**
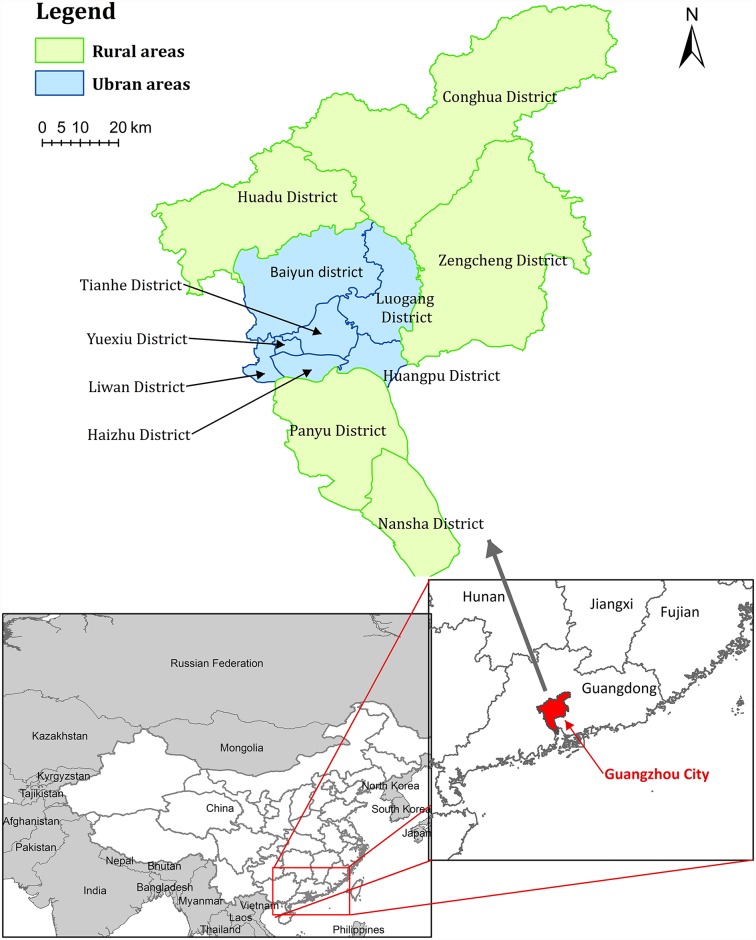
Geographic location of Guangzhou city, Guangdong province, China.

Southern China has the highest scrub typhus incidence in the country. In recent years, the illness has become an epidemic in the region. Guangzhou, the largest city in southern China, is the only city in China listing scrub typhus as a notifiable infectious disease. It reported the highest incidence of scrub typhus in the country, with an incidence 14.5 times the national average in 2012. The yearly number of scrub typhus cases were 470, 520, 1026, 884 and 938 between 2010 and 2014, with incidence rates of 45.5, 40.9, 80.5, 69.6 and 73.1 per million, respectively. The region features a subtropical monsoon climate, with an annual average relative humidity of 75% and an average temperature of 22°C. The geographic location of Guangzhou city is shown in Fig 1.

### Data summary

Data on the monthly notified scrub typhus cases in Guangzhou from 2006 to 2014 were extracted from the Chinese communicable disease network. The network covers all hospitals and community health centers in Guangzhou. When scrub typhus is suspected in a patient, the doctor is required to report it in the system. Then, the CDC conducts an epidemiological investigation and confirms the diagnosis with laboratory tests. The criteria for a clinically diagnosed case of scrub typhus include (i) field exposure history 1–3 weeks prior to symptoms, (ii) fever with skin rash or lymphadenopathy, and (iii) typical eschars or ulcers. The case definition of a laboratory-confirmed case must fulfill the above criteria and also meet at least one of the following laboratory criteria for diagnosis: a 4-fold or greater rise in serum IgG antibody titers between acute and convalescent sera-detected using an indirect immune fluorescence antibody assay (IFA), detection of *O*. *tsutsugamushi* by polymerase chain reaction (PCR) in clinical specimens, or isolation of *O*. *tsutsugamushi* from clinical specimens[8]. Our analyses were based on the laboratory-confirmed cases.

Data on the monthly rodent density were obtained through rodent population density investigation. Rodent hosts of *O*. *tsutsugamushi* were trapped in Guangzhou every month for three consecutive nights from 2006 to 2014. More than 100 traps were placed at the trapping site each night and checked each morning. Traps were placed every 5 meters in each row with at least 20 meters between rows. Rodent density was calculated as the number of rodents captured divided by the number of rodent traps.

Local climate data on monthly land surface temperature (LST), relative humidity (RH), rainfall, and diurnal temperature range (DTR) for the study period were obtained from the Chinese Bureau of Meteorology (http://data.cma.cn/). The multivariate ENSO index (MEI) was obtained from the Earth System Research Laboratory Physical Sciences Division [19]. The MEI was used as an indicator of global climate variability on the six main observed variables over the tropical Pacific. These six variables are sea-level pressure (P), zonal (U) and meridional (V) components of the surface wind, sea surface temperature (S), surface air temperature (A), and total cloud fraction of the sky (C).

### Informed consent and ethical issues

We obtained written informed consent from all study subjects. This study was reviewed and approved by the Guangzhou Center for Disease Control and Prevention Ethics Committee and the Guangzhou Health Bureau.

### Wavelet analysis

Wavelet analysis can investigate and quantify the temporal evolution of the periodic components of a time series. Wavelet analyses have been increasingly used to analyze various human infectious disease dynamics such as HFRS, measles [12], influenza [13], leishmaniasis[14] and dengue[15, 16]. We conducted a wavelet analysis on a time series of reported scrub typhus cases to detect and quantify variability of the incidence over time. In this study, the square roots of monthly scrub typhus incidences were normalized, and the trend was suppressed before analysis [9]. Wavelet coefficients (ranging from -20 to 20) were calculated in every assumed period (from 0 to 30). The result was shown in a color picture, where blue represents the valley of the trend, red represents the peak of the trend, and white indicates that no upward or downward trend was discovered. Wavelet time series analysis was applied using well-established algorithms[18] implemented in MATLAB software (MathWorks, Inc.).

### Statistical analysis

A time-series Poisson regression model allowing for over dispersion and distributed lag nonlinear models [20] was used after controlling for seasonality, long-term trends, autocorrelation, and lag effects. The models for each variable were as follows:

$$Y_{t}\sim Poisson\left(\mu \right)=\alpha +\beta_{1}X_{t,l}+S\left(COV_{t}\right)+Year_{t}+Month_{t}$$

where *t* = month of observation; *Y*_*t*_ = the number of scrub typhus cases during month *t*; α = intercept; *l* = lag months; *X*_*t*,*l*_ = cross-basis matrix of determinant and lag; *S(COV)* = cubic spline of covariates; *Year* and *Month* = indicator variables to control for long-term trends and seasonality.

We used a natural cubic spline–natural cubic spline DLNM that modeled both the nonlinear effect and the lagged effect of the determinants. The df (knots) for determinants and lag were chosen by the Akaike Information Criterion (AIC) for quasi-Poisson models[20].

Associations between determinants and scrub typhus cases were presented as relative risks (RRs) and associated 95% confidence intervals (95% CIs). All statistical tests were two-sided, and values of p < 0.05 were considered statistically significant. Analyses were performed using the *dlnm* package of R (version 3.1.1 R Development Core Team 2014) [21].

## Results

**Fig 2:**
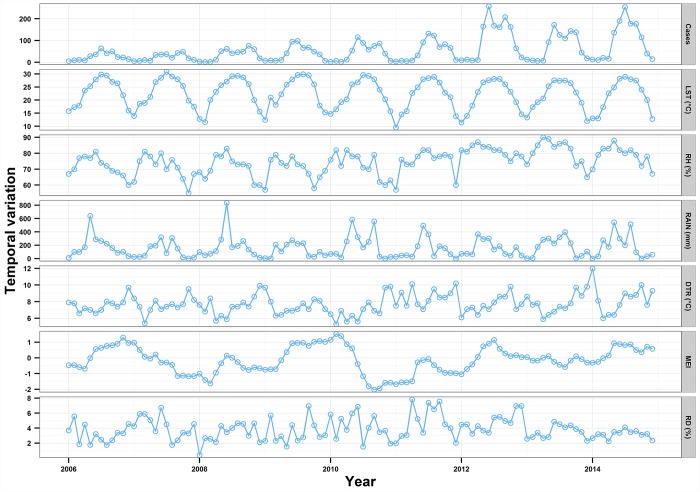
Temporal variation in the number of scrub typhus cases, climate variables, and rodent density in Guangzhou, Southern China, 2006–2014.

During the period of 2006–2014, a total of 4795 scrub typhus cases were reported in Guangzhou, with an annual average incidence of 5.40 per 100,000. On average, there were 1.6 (SD: 2.2) new cases of scrub typhus per day. Of all patients, 48.44% (2323/4795) were male and 51.56% (2472/4795) were female. The male-to-female ratio was 0.94:1. The greatest number of patients were in the ≥70 years age group, which accounted for 71.67% (2849) of all patients. In terms of occupation, 54.01% (2590) of the total cases occurred in farmers. Fig 2 and Table 1 shows the temporal variation in the number of cases, climate variables and rodent density during the study period.

**Table 1:** Descriptive statistics for daily scrub typhus cases and climate variables, in Guangzhou, Southern China, 2006–2013.

Variables	Total	Mean	SD	Min	Median	Max
No. of cases of scrub typhus	4795	1.6	2.2	0	1	17
Climate variables						
Land surface temperature (°C)	–	22.4	6.3	5.1	23.9	33.5
Relative humidity (%)	–	74.1	13.0	25.0	76.0	100.0
Rainfall (mm)	–	5.2	14.3	0.0	0.0	214.7
Diurnal temperature range (°C)	–	7.6	2.9	1.0	7.6	17.6
MEI (Standardized, monthly data)	–	-0.2	0.8	-2.0	-0.2	1.5
Rodent density (%, monthly data)	–	3.9	1.6	0.4	3.7	7.8

**Fig 3:**
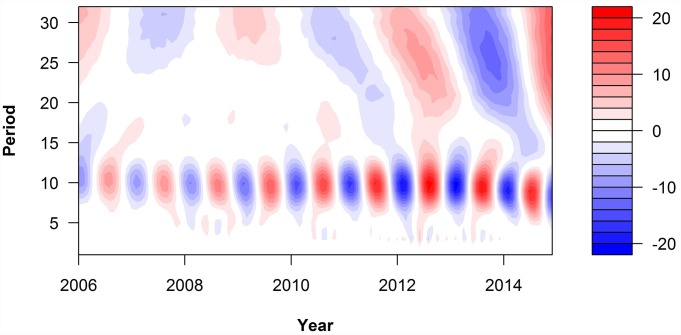
The evolution of the periodic components of scrub typhus over time.

We observed a summer peak in June and July and a second peak in September and October except in years 2009 and 2011 (Fig 2). Two main temporal cycles in the incidence of scrub typhus were discovered: a short-term trend with a period of approximately 8–12 months and long-term trend with a period of approximately 24–36 months. Timing of the breakpoints correspond with apparent changes in both size of the epidemic and patterns of seasonal variability, with appearance of the 1-year cycle in the wavelets spectrum. When the peaks of the two trends met, it showed a sharp rise in number of cases, such as in the summer of 2012 (Fig 3).

**Fig 4:**
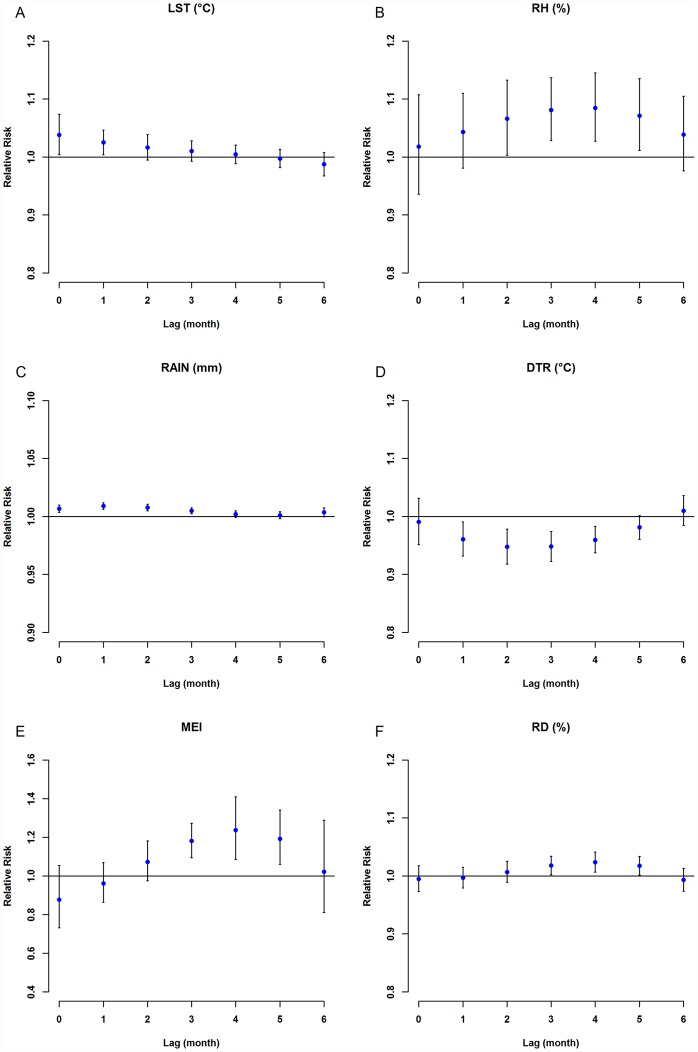
Lag-response between climate variables, including (A) land surface temperature, (B) relative humidity, (C) rainfall, and (D) diurnal temperature range and scrub typhus incidence over an 83-day period. The red lines are mean relative risks, and gray regions are 95% CIs.

**Fig 5:**
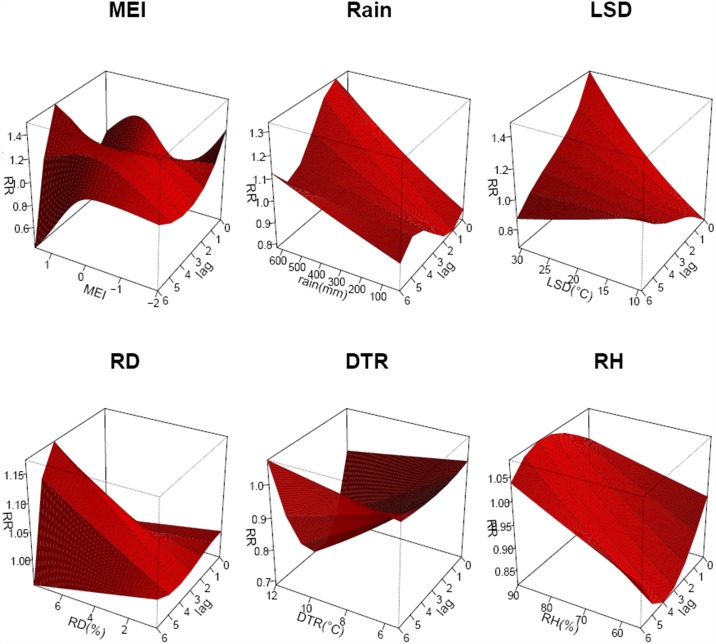
The three dimensional relationships of land surface temperature, relative humidity, rainfall, and diurnal temperature range (x-axis), lag (z-axis) and relative risk of scrub typhus incidence (y-axis).

Fig 4 shows estimated single-month lag effects of LST, RH, rainfall, DTR, MEI and RD. A 1°C increase in mean temperature was associated with a 3.8% [95% confidence interval (CI): 0.4–7.4%] increase in the risk ratio of scrub typhus cases during the same week. A 10% increase in relative humidity was associated with an 8.5% (95%CI: 2.7–14.5%) increase in the odds of scrub typhus cases after a 4-month lag. A 10-mm increase in rainfall was associated with a 0.9% (95%CI: 0.6–1.2%) increase in the odds of scrub typhus cases after a 4-month lag. A 1°C increase in the DTR was associated with a 5.3% decrease (95% CI: -7.8–-2.6%) in scrub typhus cases after a 7-week lag. A 1-unit increase in MEI was associated with a 23.6% (95% CI: 8.6–40.8%) increase in the odds of scrub typhus cases after a 5-month lag. A 1% increase in rodent density was associated with a 2.4% (95% CI: 0.7–4.1%) increase in the odds of scrub typhus cases after a 5-month lag. Fig 5 presents the 3D graph of the above factor effects.

## Discussion

Scrub typhus was endemic in China for many decades. It was reported that 28 out of 34 provinces in China were affected by scrub typhus at some point [22]. Guangzhou was also affected by scrub typhus, our study showed that from 2006 to 2014, the number of reported cases per year increased from 261 to 735. In 2012, over one thousand cases were reported. Wavelet analysis showed that there are two independent trends in the incidence of scrub typhus: a short-term trend (approximately 8–12 months) and a long-term trend (approximately 24–36 months). The short-term trend may be caused by the weather cycle and the lifecycle of the mite that carries the bacteria. The long-term trend may influenced by climate change or population mobility. When the two cycles show an upward trend at the same time, the incidence of scrub typhus grows rapidly; this may be why the number of cases in 2012 was approximately double what it was in 2011.

Human health is influenced by climate through various pathways, from the spread of pathogens to a direct effect on the immune system. Climate also plays an important role in the transmission of infectious diseases, especially vector-borne diseases, such as scrub typhus, dengue fever and hemorrhagic fever with renal syndrome (HFRS). It can affect many aspects of the rodent hosts, both individual life histories and population dynamics [16, 23]. Furthermore, due to its life-long influence on the host, human incidence does not always fluctuate immediately in response to a change in climate; there is a period of lag and accumulation. Research on the lag and accumulation effects of climate change can help predict and prevent disease outbreaks.

Temperature can influence the occurrence of human infection by inhibiting or promoting chigger activity. Our study shows that a 1°C increase in mean temperature was associated with a 3.8% increase in the odds of scrub typhus cases during the same week. Warmer conditions can be a useful predictor of scrub typhus because they can directly influence human outdoor activity and the abundance and distribution of rodents. However, some reports, such as those in India and Korea, showed a negative relationship between temperature and the incidence of scrub typhus [24, 25]. This may be because of different dominant strains or the non-linear relationship between temperature and incidence. The reason that temperature causes different effects on scrub typhus incidence requires additional research.

Relative humidity and rainfall are significant factors that affect the incidence of scrub typhus and other rodent-borne diseases. Relative humidity can affect the infectivity and stability of the mite. Our study showed that a 10% increase in relative humidity was associated with an 8.5% increase in the odds of scrub typhus cases after a 4-month lag. This may be because a high relative humidity provides a moist condition for the mite to thrive [26]. A study in Chile revealed that chiggers survive and reproduce well at a relative humidity above 50% but decrease in number or activity when relative humidity is below 50% [21]. The effect of rainfall on rodent-borne infectious diseases can be explained by the increase in vegetation as rainfall increases, which directly or indirectly makes survival and reproduction easy for rodents and causes a high rodent density [27].

MEI reflects more global patterns rather than local climate factors (such as temperature and wind) and can be more helpful in understanding long-term trends of scrub typhus. As an indicator of global climate, MEI contains six different main variables observed over the tropical Pacific. When the larger geographic area climate variables were considered in the analysis, co-linearity problems were avoided and trends became more reasonable [19, 28, 29]. Our study showed that between 2006 and 2014, a 1-unit increase in MEI was associated with a 23.6% increase in the odds of scrub typhus after a 5-month lag. In contrast to the effect of local climate factors on biological components of disease transmission, MEI could influence the environment and alter human population characteristics, such as susceptibility [30].

The rodent is an import intermediate host for the transmission of *Oriental tsutsugamushi* [31]. The density and distribution of mites can directly influence the incidence of scrub typhus. Lee IY and his team found that the high incidence of scrub typhus in humans from October to December in Korea is due to high populations of *L*. *pallidum* and *L*. *scutellare* at the same time. Additionally, the low incidence from December through August can be attributed to limited mite growth and reproduction during that period [9]. In Guangzhou, seasonal distribution of scrub typhus is associated with the local appearance of *L*. *deliense*, the dominant mite in this area. Scrub typhus is most prevalent from May to October with a peak occurrence between June and August.

To our knowledge, this study was the first to use wavelet analysis to reveal epidemiological characteristics of scrub typhus, and this is the first time that MEI was considered as a risk factor for scrub typhus. Additionally, only a few studies analyzed both climate factors and the animal reservoir in a comprehensive model to explain their effects on the incidence of scrub typhus. However, our study still has some limitations. First, the MEI data and rodent density we obtained were recorded monthly. Even though the daily incidence and other climate data were available, relationships could only be established on a monthly basis, making it impossible to evaluate the exact number of lag days. Second, the interactions of climate factors were not analyzed in this study. Finally, the incidence of scrub typhus may also be influenced by socioeconomic factors, such economy, distribution of health resources and the average resident’s medical knowledge. These limitations should be investigated in further studies.

## Supporting information

S1 Data Data on the monthly notified scrub typhus cases, LST, RH, rainfall, DTR, rodent density and MEI. (CSV)
